# Investigation of phase II metabolism of 11-hydroxy-Δ-9-tetrahydrocannabinol and metabolite verification by chemical synthesis of 11-hydroxy-Δ-9-tetrahydrocannabinol-glucuronide

**DOI:** 10.1007/s00414-020-02387-w

**Published:** 2020-08-17

**Authors:** Christoph Hassenberg, Florian Clausen, Grete Hoffmann, Armido Studer, Jennifer Schürenkamp

**Affiliations:** 1 Department of Forensic Toxicology, Institute of Legal Medicine, University Hospital Münster, Röntgenstr, 23, 48149 Münster, Germany; 2 Organisch-Chemisches Institut, Westfälische Wilhelms-Universität Münster, Corrensstrasse 40, 48149 Münster, Germany

**Keywords:** Cannabis, (−)-Δ-9-THC, (−)-11-OH-Δ-9-THC, Phase II metabolism, Synthesis of alcoholic (−)-11-OH-Δ-9-THC-glucuronide

## Abstract

(−)-Δ-9-tetrahydrocannabinol ((−)-Δ-9-THC) is the main psychoactive constituent in cannabis. During phase I metabolism, it is metabolized to (−)-11-hydroxy-Δ-9-tetrahydrocannabinol ((−)-11-OH-Δ-9-THC), which is psychoactive, and to (−)-11-nor-9-carboxy-Δ-9-tetrahydrocannabinol ((−)-Δ-9-THC-COOH), which is psychoinactive. It is glucuronidated during phase II metabolism. The biotransformation of (−)-Δ-9-tetrahydrocannabinol-glucuronide ((−)-Δ-9-THC-Glc) and (−)-11-nor-9-carboxy-Δ-9-tetrahydrocannabinol-glucuronide ((−)-Δ-9-THC-COOH-Glc) is well understood, which is mainly due to the availability of commercial reference standards. Since such a standardized reference is not yet available for (−)-11-hydroxy-Δ-9-tetrahydrocannabinol-glucuronide ((−)-11-OH-Δ-9-THC-Glc), its biotransformation is harder to study and the nature of the glucuronide bonding—alcoholic and/or phenolic—remains unclear. Consequently, the aim of this study was to investigate the biotransformation of (−)-11-OH-Δ-9-THC-Glc in vitro as well as in vivo and to identify the glucuronide by chemically synthesis of a reference standard. For in vitro analysis, pooled human S9 liver fraction was incubated with (−)-Δ-9-THC. Resulting metabolites were detected by high-performance liquid chromatography system coupled to a high-resolution mass spectrometer (HPLC-HRMS) with heated electrospray ionization (HESI) in positive and negative full scan mode. Five different chromatographic peaks of OH-Δ-9-THC-Glc have been detected in HESI positive and negative mode, respectively. The experiment set up according to Wen et al. indicates the two main metabolites being an alcoholic and a phenolic glucuronide metabolite. In vivo analysis of urine (*n* = 10) and serum (*n* = 10) samples from cannabis users confirmed these two main metabolites. Thus, OH-Δ-9-THC is glucuronidated at either the phenolic or the alcoholic hydroxy group. A double glucuronidation was not observed. The alcoholic (−)-11-OH-Δ-9-THC-Glc was successfully chemically synthesized and identified the main alcoholic glucuronide in vitro and in vivo. (−)-11-OH-Δ-9-THC-Glc is the first reference standard for direct identification and quantification. This enables future research to answer the question whether phenolic or alcoholic glucuronidation forms the predominant way of metabolism.

**Electronic supplementary material:**

The online version of this article (10.1007/s00414-020-02387-w) contains supplementary material, which is available to authorized users.

## Introduction

Cannabis is worldwide the most commonly abused illegal drug and contains at least 90 different phytocannabinoids. Phytocannabinoids are terpenophenolic secondary metabolites preferably produced in cannabis. The most important ones are (−)-Δ-9-tetrahydrocannabinol ((−)-Δ-9-THC), cannabidiol (CBD), cannabinol (CBN), and (−)-Δ-8-tetrahydrocannabinol ((−)-Δ-8-THC), of which (−)-Δ-9-THC is the main psychoactive component [1, 2]. Besides its abuse as a recreational drug, the importance for cannabis or THC as a therapeutic drug is growing [3], pressing the need to fully understand its metabolism within the human body.

**Fig. 1:**
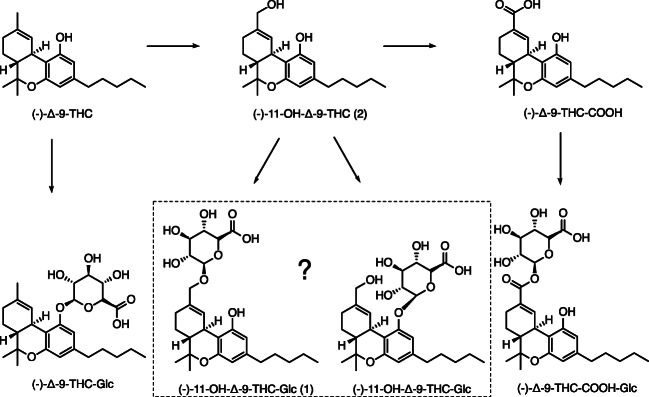
Main metabolism steps of (−)-Δ-9-THC via (−)-11-OH-Δ-9-THC and (−)-Δ-9-THC-COOH to its phase II metabolites (−)-Δ-9-THC-Glc, (−)-11-OH-Δ-9-THC-Glc, and (−)-Δ-9-THC-COOH-Glc

In the human body, over 80 metabolites of psychoactive (−)-Δ-9-THC are described [4]. During phase I metabolism (see Fig. 1), (−)-Δ-9-THC is mainly hydroxylated by cytochrome P450 2C9 (CYP2C9) and, to a minor extend, by cytochrome P450 2C19 (CYP2C19) to psychoactive (−)-11-hydroxy-Δ-9-tetrahydrocannabinol ((−)-11-OH-Δ-9-THC).

Minor hydroxy-metabolites such as 8-*α*/*β*-OH-Δ-9-THC, 8,11-diOH-Δ-9-THC, or 9,10-epoxy-Δ-9-THC are metabolized by CYP3A4 [5–8]. While this first hydroxylation is characterized in detail, the second oxidation step to (−)-Δ-9-THC-COOH via 11-oxo-Δ-9-THC is not yet equally well understood. Watanabe et al. [9] showed that in rat liver, cytochrome P450 enzymes are responsible for the microsomal aldehyde oxygenase (MALDO) activity, which catalyzes the (−)-Δ-9-THC-COOH biotransformation [7, 9–11]. In the human liver, CYP3A4 and CYP2C9 were identified to be the main enzymes for the oxidation of 11-oxo-Δ-8-THC [12]. However, there are no data available on the MALDO activity of CYP enzymes concerning 11-oxo-Δ-9-THC biotransformation.

During phase II metabolism (see Fig. 1), (−)-Δ-9-THC, (−)-11-OH-Δ-9-THC, and (−)-Δ-9-THC-COOH are glucuronidated by UDP-glucuronosyltransferases (UGT). Mazur et al. [13] identified UGT1A1 and UGT1A3 to be responsible for the glucuronidation of (−)-Δ-9-THC-COOH, whereas UGT1A9 and UGT1A10 are responsible for the glucuronidation of (−)-Δ-9-THC and (−)-11-OH-Δ-9-THC. For polymorphic UGT1A9, Schneider et al. [14] recognized significantly lower (−)-11-OH-Δ-9-THC concentrations of homozygote carriers of the derived alleles in − 440/− 331 of the UGT1A9 gene compared with homozygote carriers of the ancestral alleles. A quantification of the resulting (−)-11-OH-Δ-9-THC-glucuronide was hitherto not possible due to the lack of a suitable reference standard. In addition, it is currently only indirectly known that (−)-11-OH-Δ-9-THC-glucuronide is formed because of higher (−)-11-OH-Δ-9-THC concentrations after glucuronide cleavage [15]. It is therefore also unknown whether the alcoholic or phenolic hydroxy group, or both, is glucuronidated.

The aim of this study was to investigate the biotransformation of (−)-11-OH-Δ-9-THC-Glc in vitro as well as in vivo and to synthesize a reference standard for direct identification and quantification to fill the gap in main metabolite reference standards. For in vitro analysis, a human S9 liver fraction assay was selected, because it has proved to be a simple tool for metabolism studies for cannabinoids [9–11] and because it is used since a long time in our working group [16, 17]. Metabolite detection was performed by high-performance liquid chromatography system coupled to high-resolution mass spectrometry (HPLC-HRMS) with heated electro spray ionization (HESI) in positive and negative full scan modes. To check the in vitro results, urine and serum samples of cannabis users, routinely analyzed at the Department of Forensic Toxicology Münster (Germany), are analyzed for (−)-11-OH-Δ-9-THC-glucuronide and verified with the reference standard synthesized.

## Material and methods

### Chemicals

Acetonitrile, methanol and water were all LC-MS grade and purchased from VWR (Darmstadt, Germany), formic acid from Merck (Darmstadt, Germany). (−)-Δ-9-THC, (−)-Δ-8-THC, (±)-11-OH-Δ-9-THC and (−)-Δ-9-THC-COOH were purchased from Lipomed (Weil am Rhein, Germany). 8-*β*-OH-Δ-9-THC and Δ-9-THC-Glc were purchased from ElSohly Laboratories (Oxford, USA). (+)-11-nor-9-Carboxy-Δ-9-THC glucuronide was purchased from Cerilliant (Rock Round, USA). Ammonium acetate, trimethylsilyl triflate (TMSOTf), olivetol and Li(O^*t*^Bu)_3_AlH (1.0 m in THF) were purchased from Sigma-Aldrich (Darmstadt, Germany). Methyl 2,3,4-tri-O-isobutyryl-1-O-trichloroacetimidoyl-α-d-glucopyranuronate and (−)-11-OH-Δ-9-THC (solid, **2**) were purchased from TRC, Canada. Alamethicin, d-saccharic acid 1,4-lactone monohydrate, nicotinamide adenine dinucleotide phosphate (NADPH) tetrasodium salt and uridine 5′-diphosphoglucuronic acid trisodium salt (UDPGA) were purchased from Santa Cruz Biotechnologies (Dallas, USA). Human liver S9 fraction (pooled—number of donors 34) was purchased from Thermo Fisher Scientific (Schwerte, Germany). *n*-butyl lithium (*n*-BuLi, 1.6 M in hexanes—technical mixture of *n*-hexane and iso-hexane) and molecular sieves were purchased from Acros Organics (now Fischer Scientific GmbH, Niederau, Germany). Solvents for flash chromatography (FC) were freshly distilled before use. Diethyl ether (Et_2_O) was refluxed over potassium metal and freshly distilled from metal potassium-sodium-alloy (4:1) afterwards. Tetrahydrofuran (THF) was refluxed over sodium metal and distilled from potassium metal afterwards.

### Analysis of synthesis intermediates

#### Isolation of synthesis intermediates

Flash chromatography was performed on Merck silica gel 60 (40–63 μm) with an excess argon pressure up to 1.0 bar. Merck silica gel 60 F254 plates were used for thin-layer chromatography (TLC) using UV light (254/366 nm), KMnO_4_ (1.5 g in 200 mL H_2_O, 5 g NaHCO_3_) for detection.

#### NMR analysis of synthesis intermediates

^1^H NMR (500 MHz and 600 MHz) and ^13^C NMR (125 MHz and 151 MHz) spectra were measured on an Agilent DD2 500 or an Agilent DD2 600 spectrometer (Waldbronn, Germany). The multiplicity of all signals was described as s (singlet), d (doublet), t (triplet), q (quartet), p (pentet), h (hextet), hept (heptet) and m (multiplet). Chemical shifts (δ in ppm) were referenced on the residual peak of CDCl_3_ (^1^H NMR: δ = 7.26; ^13^C NMR: δ = 77.0), C_6_D_6_ (^1^H NMR: δ = 7.16; ^13^C NMR: δ = 128.06) or CD_3_OD (^1^H NMR: δ = 3.31; ^13^C NMR: δ = 49.00).

#### MS analysis of synthesis intermediates

HRMS ESI measurements were performed using a Bruker MicroTof (Bremen, Germany).

### Synthesis of (−)-11-OH-Δ-8-THC-Glc (11)

#### Synthesis of ((1R,5S)-4-hydroxy-6,6-dimethylbicyclo[3.1.1]hept-2-en-2-yl)methyl-pivalate (**6**)

In an inert atmosphere, (1*S*,5*R*)-4-(hydroxymethyl)-6,6-dimethylbicyclo[3.1.1]hept-3-en-2-on (**5**) (0.72 g, 4.3 mmol, 1.0 equiv.) was dissolved in CH_2_Cl_2_ (10 mL) at 0 °C and pyridine (0.52 g, 0.53 mL, 6.5 mmol, (1.5 equiv.) was added. Subsequently, pivaloyl chloride (0.79 g, 0.80 mL, 6.5 mmol, 1.5 equiv.) was added and the reaction mixture was stirred for 2 h. The volatile solvents were removed under reduced pressure and the crude product was dissolved in THF (20 mL) followed by the dropwise addition of Li(O^*t*^Bu)_3_AlH (1.0 m in THF, 5.3 mL, 5.3 mmol, 1.5 equiv.). The solution was stirred over night while letting warm to room temperature (rt., 20 °C). An aqueous solution of NaHCO_3_ was added and the reaction mixture was extracted with ether. The organic extract was dried over MgSO_4_ and the solvent was removed under reduced pressure. After FC (pentane:ethyl acetate, 5:1 (v:v)), the product was isolated as a yellow oil (0.77 g, 3.1 mmol, 70% yield).

#### Synthesis of tert-butyl-2-((6aR,10aR)-1-hydroxy-6,6-dimethyl-3-pentyl-6a,7,10,10a-tetrahydro-6H-benzo[c]chromen-9-yl) pivalate (**7**)

In analogy to a procedure by Hoffmann and Studer [18, 19], ((1*R*,5*S*)-4-hydroxy-6,6-dimethylbicyclo[3.1.1]hept-2-en-2-yl)methyl-pivalate (**6**) (2.5 g, 9.9 mmol, 1.5 equiv.) and olivetol (1.2 g, 6.6 mmol, 1.0 equiv.) were dissolved in CH_2_Cl_2_ (50 mL) in an inert atmosphere and cooled to − 20 °C. HBF_4_·OEt_2_ (3.4 mL, 27 mmol, 4.0 equiv.) was slowly added dropwise and the reaction mixture stirred for 2 h. The temperature was raised to rt. in the course of 1 h and an aqueous solution of NaHCO_3_ was added to the flask in one portion. The reaction mixture was extracted with ethyl acetate, and the organic extract was dried over MgSO_4_ followed by removal of the solvent under reduced pressure. After FC (pentane:ethyl acetate, 15:1 (v:v)), the product was isolated as a colorless oil (0.77 g, 1.9 mmol, 28% yield).

#### Synthesis of (−)-11-OH-Δ-8-THC (**8**)

In an inert atmosphere, ((6a*R*,10a*R*)-1-hydroxy-6,6-dimethyl-3-pentyl-6a,7,10,10a-tetrahydro-6H-benzo[c]chromen-9-yl)methyl pivalate (**7**) (0.75 g, 1.8 mmol, 1.0 equiv.) was dissolved in THF and the solution was cooled to 0 °C before the stepwise addition of LiAlH_4_ (0.28 g, 7.2 mmol, 4.0 equiv.) and subsequent stirring for 3 h. While cooling on ice, water was carefully added to the reaction and the mixture was extracted with ether. The organic extract was dried over MgSO_4_ and the solvent removed under reduced pressure. After FC (pentane:ethyl acetate, 2:1 (v:v)), the product was isolated as a colorless solid. (0.47 g, 1.4 mmol, 79% yield).

#### Synthesis of (−)-11-OH-Δ-8-THC-OAc (**9**)

In an inert atmosphere, a solution of (−)-11-OH-Δ-8-THC (**8**) (20 mg, 61 μmol, 1.0 equiv.) in ethyl acetate (2 mL) was cooled to 0 °C before triethylamine (9.0 mg, 13 μL, 90 μmol, 1.5 Äquiv.) and acetyl chloride (7 mg, 6 μL, 9 μmol, 1.5 Äquiv.) were added in that sequence. The mixture was stirred for 1 h and water was added. The reaction mixture was extracted with ethyl acetate and the organic extract was dried over MgSO_4_ followed by removal of the solvent under reduced pressure. After FC (pentane:ethyl acetate, 2:1 (v:v)), the product was isolated as a colorless oil (13 mg, 35 μmol, 57% yield).

#### Synthesis of (−)-11-OH-Δ-8-THC-Glc-OAc (**10**)

In an inert atmosphere, (−)-11-OH-Δ-8-THC-OAc (**9**) (40 mg, 0.12 mmol, 1.0 equiv.) and methyl 2,3,4-tri-O-isobutyryl-1-O-trichloroacetimidoyl-α-d-glucopyranuronate (100 mg, 0.18 mmol, 1.5 equiv.) were dissolved in dichloroethane (1 mL) and cooled to − 30 °C. Afterwards, BF_3_·OEt_2_ (13 mg, 11 μL, 90 μmol, 0.75 Äquiv.) was added via a syringe and the mixture stirred for another 3 h. After warming to rt., water was added and the reaction mixture was extracted with CH_2_Cl_2_. The organic extract was dried over MgSO_4_ followed by removal of the solvent under reduced pressure. After FC (pentane:ethyl acetate, 5:1 (v:v)), the product was isolated as a colorless oil (42 mg, 6.0 μmol, 50% yield).

#### Synthesis of (−)-11-OH-Δ-8-THC-Glc (**11**)

To a solution of (−)-11-OH-Δ-8-THC-Glc-OAc (**10**) (20 mg, 30 μmol, 1.0 equiv.) in EtOH (1 mL) was added NaOH (2 M in water, 1 mL) at rt. After stirring for 24 h, water and CH_2_Cl_2_ were added and the phases separated. The aqueous phase was washed with CH_2_Cl_2_ and hydrochloric acid was added for protonation of the deprotonated product. Extraction with CH_2_Cl_2_ and subsequent removal of the solvent under reduced pressure delivered the product as colorless oil (9 mg, 15 μmol, 50% yield).

### Synthesis of (−)-11-OH-Δ-9-THC-Glc (1)

#### Synthesis of (−)-11-OH-Δ-9-THC-OAc (**12**)

Under an inert atmosphere, *n*-butyllithium (1.6 M in hexanes, 50 μL, 75 μmol, 1.0 equiv.) was added to a solution of (−)-11-OH-Δ-9-THC (**2**) (25 mg, 75 μmol, 1.0 equiv.) in THF (2.5 mL) at − 78 °C. The solution was stirred for 1 h before warmed to rt. and acetic anhydride (9.2 mg, 8.5 μL, 90 μmol, 1.2 equiv.) was added. The resulting mixture was stirred for another 12 h. The solvent was removed under reduced pressure and after separation by FC (0.5% MeOH in CH_2_Cl_2_), the product **12** was obtained as a colorless oil (26 mg, 69 μmol, 90% yield).

#### Synthesis of (−)-11-OH-Δ-9-THC-Glc-OAc (**13**)

Under an inert atmosphere, TMSOTf (0.7 μL) was added to a suspension of (−)-11-OH-Δ-9-THC-OAc (**12**) (4.4 mg, 12 μmol, 1.0 equiv.), methyl 2,3,4-tri-O-isobutyryl-1-O-trichloroacetimidoyl-α-d-glucopyranuronate (14 mg, 24 μmol, 2.0 equiv.) and powdered molecular sieves (4 Å, 20 mg) in CH_2_Cl_2_ (1 mL) at 0 °C. After 1 h, another portion of TMSOTf (0.7 μL) was added and the reaction mixture further stirred for 1 h. The solvent was removed under reduced pressure and after separation by FC (0.5% MeOH in CH_2_Cl_2_), the product **13** was obtained as colorless oil (3.4 mg, 4.4 μmol, 37% yield).

#### Synthesis of (−)-11-OH-Δ-9-THC-Glc (**1**)

To a solution of (−)-11-OH-Δ-9-THC-Glc-OAc (**13**) (5.0 mg, 6.4 μmol, 1.0 equiv.) in H_2_O (1 mL) and MeOH (1 mL) was added LiOH (50 mg, 2.1 mmol, 325 equiv.) at rt. After stirring for 24 h, methanol was removed under reduced pressure. The aqueous phase was washed with ether (2 × 2 mL) and acetic acid (1 mL) was added for protonation of the deprotonated product. Extraction with ether (3 × 2 mL) and subsequent removal of the solvent under reduced pressure delivered the product as a colorless solid (3.1 mg, 6.1 μmol, 95% yield).

### In vitro analysis

The assay used for in vitro analysis is a combination of two previously published and in our working group established S9 fraction assays (Schwarzkopf et al. [16] and Holtfrerich et al. [17]). The combined assay was checked with respect to detergent use, incubation time and amount of protein used (data not shown). To verify the qualitative assay, paracetamol, as a standard test substance used in the working group, was first incubated with pooled human liver S9 fraction. Finally, (−)-Δ-9-THC reference solution and a negative control (phosphate buffer instead of NADPH/UDPGA solution) were incubated (*n* = 1, respectively) with the following assay details:

All solutions, unless otherwise specified, were prepared in 0.1 M phosphate buffer. Final concentrations are given in brackets. To a solution of 5 μL alamethicin (100 μg/mL in EtOH/H_2_O, 1:1, v:v), 125 μL of pooled human liver S9 fraction (2 mg protein/mL) was added and then incubated for 15 min on ice. After that, 72 μL of 0.1 M phosphate buffer, 100 μL saccharolactone solution (4.9 mM), 100 μL of MgCl_2_ solution (1.9 mM) and 3.14 μL of (−)-Δ-9-THC solution (1 mg/mL in EtOH/H_2_O, 1:1, v:v, 19.7 μM) are added sequentially. After incubating for 3 min at 37 °C in a water bath, 100 μL of NADPH/UDPGA solution (0.9 mM NADPH/4.9 mM UDPGA) was added, leading to 505.14 μL total volume. The final EtOH concentration was 0.8%vol. Then, the mixture was incubated for 60 min at 37 °C. The reaction was stopped with 200 μL ice-cold acetonitrile and the mixture was cooled at 0 °C for 15 min. After 1 min vortexing and 5 min centrifugation at 12,000×*g*, 100 μL supernatant was removed, diluted with 50 μL methanol, and applied to HPLC-MS/MS analysis.

### In vivo analysis

To verify the in vitro results, 10 urine samples and 10 serum samples from cannabis users, routinely analyzed at the Department of Forensic Toxicology Münster (Germany), were investigated. The urine sample (50 μL) was diluted 1:10 (v:v) by adding 200 μL 2 mM ammonium acetate buffer containing 0.1% formic acid and 250 μL acetonitrile with 0.1% formic acid. After centrifugation for 10 min at 12,000×g, 2 μL was injected into the HPLC-HRMS in full scan MS-1 mode. The serum samples were analyzed by solid phase extraction (SPE) followed by HPLC-HESI(+)-HRMS in parallel reaction monitoring (PRM) mode, because of its higher sensitivity compared with full scan mode. Five hundred microliters of serum was diluted with phosphate buffer (pH 6) and internal standard was added. Before loading, the SPE-C18 cartridge was activated in a common way. Successively, the SPE was washed with H_2_O, dried under vacuum and the analytes were eluted in two steps with acetone and methanol containing 0.1% formic acid. Then, the extracts were evaporated and resolved in 100 μL volume to achieve a higher sensitivity. The injection volume was 10 μL. The chromatographic separation was achieved with a gradient of 2 mM ammonium acetate buffer containing 0.1% formic acid and methanol with 0.1% formic acid (manuscript in preparation).

### HPLC-HRMS and HPLC-HRMS/MS method

Metabolite screening was achieved by analyzing the abovementioned in vitro and in vivo samples with a Thermo Fisher Scientific UltiMate 3000 HPLC system coupled with a Q Exactive Focus™ Quadrupole-Orbitrap Mass Spectrometer (Thermo Scientific, Bremen, Germany). For chromatographic separation, a Thermo Acclaim™ (120 C18 3 μm 120 Å 2.1 × 100 mm) phase was used at 40 °C with a multistep gradient (eluent A was 2 mM ammonium acetate buffer containing 0.1% formic acid and eluent B acetonitrile with 0.1% formic acid). Initially, 50% B was kept for 0.5 min, then raised to 95% until 9 min, kept for 2 min at 95% B, decreased to 50% B within 0.1 min and kept until 14 min for equilibration. A flow rate of 300 μL/min and an injection volume of 2 μL were used. Ionization was achieved with a heated electro spray (HESI) in switching mode. The source parameters were as follows: auxiliary gas flow rate, sheath gas flow rate, sweep gas flow rate: 11, 2 and 48 arbitrary units, respectively; auxiliary gas heater 413 °C; capillary temperature 256 °C; and spray voltage ± 3.5 kV. The mass spectrometer was operated in full MS-1 mode with a mass range from *m*/*z* 200 to 1000 and resolution of 70,000 (full width at half maximum (FWHM) at *m*/*z* 200). The automatic gain control (AGC) target was set to 1 × 10^6^ and the maximum injection time to “auto”. Data were recorded in profile data format.

For dd-MS-2 mode, the resolution was set to 35,000 (full width at half maximum (FWHM) at *m*/*z* 200), AGC target to 2 × 10^5^ and max. injection time to “auto.” Data were recorded in profile data format. The isolation window was set to 1.5 *m*/*z*. For fragmentation, the normalized fragmentation energy was set to 40 eV.

## Results and discussion

### Synthesis strategy

The synthesis educt (−)-11-OH-Δ-9-THC (2) is commercially available but very cost-intensive. For this reason, only a very small amount (50 mg) was available for synthesis of (−)-11-OH-Δ-9-THC glucuronide. Since, until today, there was no synthesis specification for the glucuronidation of (−)-11-OH-Δ-9-THC (2), this was too little starting substrate for method development. Therefore, in a first step, the synthetically more easily accessible (−)-11-OH-Δ-8-THC was produced in larger quantities. The glucuronidation reaction at the alcoholic hydroxy group was then developed on this (−)-11-OH-Δ-8-THC educt and transferred to less available (−)-11-OH-Δ-9-THC educt.

### Synthesis of (−)-11-OH-Δ-8-THC-Glc (11)

**Fig. 2:**
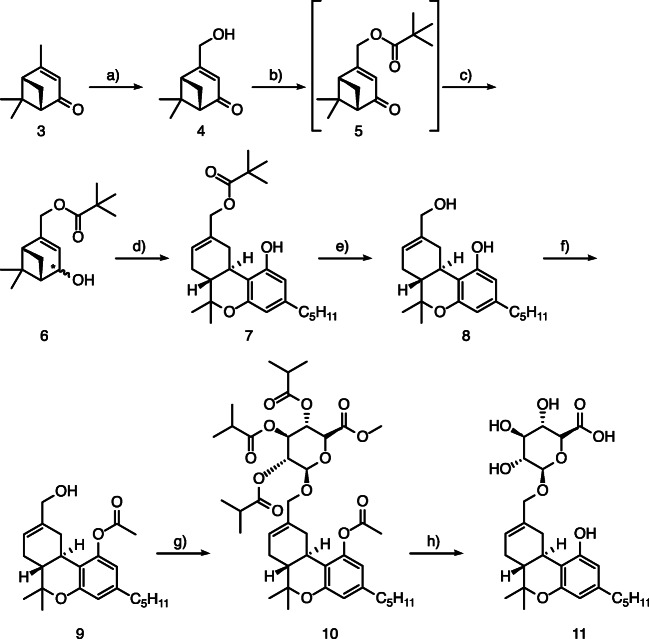
Synthesis of (−)-11-OH-Δ-8-THC-Glc (11) starting from (−)-verbenone (3): (a) see reference [20], (b) pyridine, pivaloyl chloride, CH_2_Cl_2_, 0 °C, 2 h; (c) lithium tri-*tert*-butoxyaluminum hydride, THF, 0 °C, overnight; (d) olivetol, tetrafluoroboric acid diethyl ether complex, − 20 °C to rt., 1 h; (e) lithium aluminum hydride, THF, 0 °C, 3 h; (f) triethylamine, acetyl chloride, EtOAc, 0 °C, 1 h; (g) methyl 2,3,4-tri-O-isobutyryl-1-O-trichloroacetimidoyl-α-d-glucopyranuronate, boron trifluoride diethyl etherate, 1,2-dichloroethane, − 30 °C, 3 h; (h) NaOH, EtOH/H_2_O, rt., 24 h

We started the synthesis of (−)-11-OH-Δ-8-THC-Glc (11), (2S,3S,4S,5R,6S)-3,4,5-trihydroxy-6-(((6aR,10aR)-1-hydroxy-6,6-dimethyl-3-pentyl-6a,7,10,10a-tetrahydro-6H-benzo[c]chromen-9-yl)methoxy)tetrahydro-2H-pyran-2-carboxylic acid (Fig. 2), with the commercially available (−)-verbenone (3), which was transformed to the alcohol 4 following a previously published procedure [20]. This alcohol was further reacted to the intermediate ketone 5 and subsequently reduced to the alcohol 6 as a mixture of diastereomers in 70% yield. For the construction of the THC-core-structure, a slightly modified version of the method developed by Hoffmann and Studer [18] and Hoffmann et al. [19] was used. Alcohol 6 and olivetol in large excess were converted into the OH-11-pivaloyl-protected THC-derivative 7 with the use of HBF_4_·OEt_2_ as the activating LEWIS acid in 28% yield. Subsequent deprotection under reductive reaction conditions delivered (−)-11-OH-Δ-8-THC (8) in 79% isolated yield. A selective acetyl protection of the phenolic hydroxyl group was obtained by a mixture of triethylamine and acetyl chloride at low temperature (0 °C), and the desired protected product 9 was isolated in 57% yield. For the glucuronidation of (−)-11-OH-Δ-8-THC (9), a previously published procedure [21, 22] was applied and 10 was obtained in 50% yield. Deprotection of the protecting groups with sodium hydroxide finally led to the (−)-11-OH-Δ-8-THC-Glc (11) in 50% yield.

#### Synthesis of ((1R,5S)-4-hydroxy-6,6-dimethylbicyclo[3.1.1]hept-2-en-2-yl)methyl-pivalate (**6**)

1H NMR (600 MHz, CDCl3, 299 K) δ = 5.66–5.64 (m, 1H), 4.53–4.44 (m, 3H), 2.52–2.47 (m, 1H), 2.35–2.30 (m, 1H), 2.10 (td, J = 5.5, 1.4 Hz, 1H), 1.36 (s, 3H,), 1.35 (d, J = 10.0 Hz, 1H), 1.20 (s, 9H), 1.08 (s, 3H). 13C NMR (150.73 MHz, CDCl3, 299 K) δ = 178.4, 145.6, 122.1 73.2, 65.8, 45.5, 44.2, 39.1, 39.0, 35.9, 27.4, 26.8, 23.0. HRMS (ESI) *m*/*z*: 275.1618 calcd. for C_16_H_25_NO_2_Na+ [M+Na]^+^, found 275.1633.

#### Synthesis of *tert*-butyl-2-((6aR,10aR)-1-hydroxy-6,6-dimethyl-3-pentyl-6a,7,10,10a-tetrahydro-6H-benzo[c]chromen-9-yl) pivalate (7)

1H NMR (600 MHz, CDCl3, 299 K) δ = 6.28 (d, J = 1.5 Hz, 1H), 6.10 (d, J = 1.5 Hz, 1H), 5.75 (d, J = 4.8 Hz, 1H), 4.70 (s, 1H), 4.50 (s, 2H), 3.37–3.33 (m, 1H), 2.73–2.68 (m, 1H), 2.44 (td, J = 7.5, 3.2 Hz, 2H,), 2.24 (d, J = 15.6 Hz, 1H), 1.88–1.81 (m, 3H), 1.60–1.56 (m, 2H), 1.38 (s, 3H), 1.33–1.29 (m, 4H), 1.27–1.25 (m, 1H), 1.22 (s, 9H), 1.11 (s, 3H), 0.88 (t, J = 7.0 Hz, 3H). 13C NMR (150.73 MHz, CDCl3, 299 K) δ = 178.4, 154.8, 154.8, 142.9, 134.0, 123.3, 110.1, 110.0, 107.6, 76.5, 68.0, 44.8, 38.9, 35.4, 31.7, 31.6, 31.3, 30.6, 27.7, 27.5, 27.2, 22.5, 18.4, 14.0. HRMS (ESI) *m*/*z*: 437.2662 calcd. for C_26_H_38_O_4_Na+ [M+Na]^+^, found 437.2639.

#### Synthesis of (−)-11-OH-Δ-8-THC (8)

1H NMR (600 MHz, CDCl3, 299 K) δ = 6.64 (d, J = 1.6 Hz, 1H), 6.01 (d, J = 1.6 Hz, 1H), 5.47 (s, 1H), 3.90–3.84 (m, 2H), 3.73 (dd, J = 17.1, 6.2 Hz, 1H), 2.81 (td, J = 11.2, 4.6 Hz, 1H), 2.46–2.44 (m, 2H), 1.89–1.86 (m, 2H), 1.83–1.78 (m, 1H), 1.59–1.56 (m, 3H), 1.30 (s, 3H), 1.30–1.25 (m, 4H), 1.00 (s, 3H), 0.86–0.84 (m, 3H). 13C NMR (150.73 MHz, CDCl3, 299 K) δ = 156.0, 155.7, 142.9, 138.7, 128.4, 121.7, 110.8, 110.5, 108.1, 76.3, 67.3, 45.6, 36.0, 32.0, 31.9, 31.3, 30.5, 27.8, 23.0, 18.5, 14.3. HRMS (ESI) *m*/*z*: 353.2087 calcd. for C_21_H_30_O_3_Na+ [M+Na]^+^, found 353.2089.

#### Synthesis of (−)-11-OH-Δ-8-THC-OAc (9)

1H NMR (600 MHz, CDCl3, 299 K) δ = 6.56 (d, J = 1.7 Hz, 1H), 6.42 (d, J = 1.7 Hz, 1H), 5.73 (d, J = 5.2 Hz, 1H), 4.07–3.99 (m, 2H), 3.00 (dd, J = 16.1, 4.6 Hz, 1H), 2.60 (td, J = 11.1, 4.6 Hz, 1H), 2.50 (td, J = 7.5, 2.7 Hz, 2H), 2.29 (s, 3H), 2.24–2.20 (m, 1H), 1.93–1.86 (m, 2H), 1.83–1.81 (m, 1H), 1.61–1.56 (m, 2H), 1.39 (s, 3H), 1.33–1.30 (m, 4H), 1.11 (s, 3H), 0.88 (t, J = 7.0 Hz, 3H). 13C NMR (150.73 MHz, CDCl3, 299 K) δ = 169.2, 154.6, 149.9, 143.1, 137.8, 121.8, 115.8, 115.5, 114.6, 76.9, 67.1, 45.0, 35.5, 31.8, 31.7, 31.7, 30.59, 27.7, 27.6, 22.8, 21.4, 18.6, 14.2. HRMS (ESI) *m*/*z*: 395.2193 calcd. for C_23_H_32_O_4_Na+ [M+Na]^+^, found 395.2185.

#### Synthesis of (−)-11-OH-Δ-8-THC-Glc-OAc (10)

1H NMR (600 MHz, CDCl3, 299 K) δ = 6.55 (d, J = 1.7 Hz, 1H, CH), 6.38 (d, J = 1.7 Hz, 1H, CH), 5.73 (d, J = 4.0 Hz, 1H), 5.30 (t, J = 9.5 Hz, 1H), 5.22 (t, J = 9.7 Hz, 1H), 5.05 (dd, J = 9.5, 8.0 Hz, 1H), 4.55 (d, J = 8.0 Hz, 1H), 4.12 (dd, J = 21.7, 12.6 Hz, 2H), 4.02 (d, J = 9.7 Hz, 1H), 3.72 (s, 3H), 2.95 (dd, J = 15.9, 5.8 Hz, 1H), 2.56–2.44 (m, 4H), 2.37 (dt, J = 14.0, 7.0 Hz, 1H, CH), 2.29 (s, 3H, CH3), 2.24–2.19 (m, 1H), 1.845–1.76 (m, 3H), 1.59–1.51 (m, 4H), 1.39 (s, 3H), 1.31 (tt, J = 7.2, 4.1 Hz, 5H), 1.11–1.05 (m, 14H), 1.02 (dd, J = 7.0, 5.2 Hz, 4H), 0.88 (t, J = 7.0 Hz, 3H). 13C NMR (150.73 MHz, CDCl3, 299 K) δ = 175.9, 175.2, 169.2, 167.2, 154.3, 149.8, 142.9, 133.9, 124.9, 115.7, 115.2, 114.4, 99.4, 76.6, 74.1, 73.0, 71.7, 70.6, 69.3, 52.9, 44.8, 35.3, 33.8, 33.8, 33.8, 31.9, 31.5, 31.4, 30.5, 27.6, 27.3, 27.2, 22.5, 21.2, 18.8, 18.8, 18.7, 18.7, 18.7, 18.6, 18.3, 14.0. HRMS (ESI) *m*/*z*: 795.3926 calcd. for C_42_H_56_O_13_Na+ [M+Na]^+^, found 795.3949.

#### Synthesis of (−)-11-OH-Δ-8-THC-Glc (11)

1H NMR (600 MHz, CD3OD, 299 K) δ = 6.16 (d, J = 1.5 Hz, 1H), 6.08 (d, J = 1.5 Hz, 1H), 5.81 (d, J = 5.1 Hz, 1H), 4.34 (d, J = 7.8 Hz, 1H), 4.23 (d, J = 12.0 Hz, 1H), 4.06 (d, J = 12.0 Hz, 1H), 3.76 (d, J = 9.8 Hz, 1H), 3.53 (t, J = 9.1 Hz, 1H), 3.47–3.44 (m, 1H), 3.39–3.35 (m, 1H), 3.27–3.23 (m, 1H), 2.65 (dt, J = 11.0, 5.7 Hz, 1H), 2.42–2.39 (m, 2H), 2.28–2.23 (m, 1H), 1.90–1.81 (m, 2H), 1.77 (dd, J = 11.8, 4.5 Hz, 1H), 1.58–1.53 (m, 2H), 1.35 (s, 3H), 1.31 (dd, J = 15.5, 6.6 Hz, 4H), 1.08 (s, 3H), 0.90 (t, J = 7.1 Hz, 3H). 13C NMR (150.73 MHz, CDCl3, 299 K) δ = 172.5, 157.8, 155.8, 143.5, 136.2, 125.2, 111.6, 109.8, 108.5, 103.7, 102.9, 77.5, 77.2, 76.6, 74.7, 74.5, 73.2, 46.7, 36.6, 33.5, 32.9, 32.6, 32.1, 28.9, 28.0, 23.6, 18.6, 14.4. HRMS (ESI) *m*/*z*: 505.2443 calcd. for C_27_H_37_O_9_− [M−H] −, found 505.2439.

### Synthesis of (−)-11-OH-Δ-9-THC-Glc (1)

**Fig. 3:**
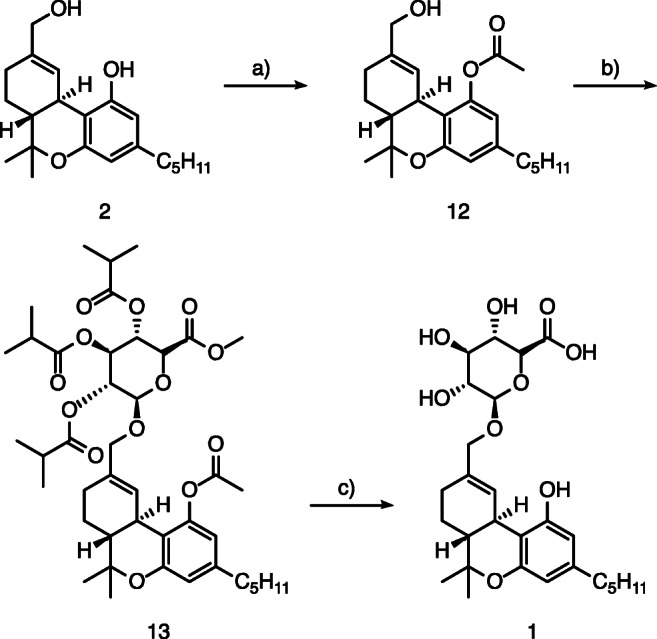
Synthesis of (−)-11-OH-Δ-9-THC-Glc (1) starting from (−)-11-OH-Δ-9-THC (2): (a) *n*-butyllithium, THF, − 78 °C to rt., 1 h; acetic anhydride, THF, rt., 12 h; (b) methyl 2,3,4-tri-O-isobutyryl-1-O-trichloroacetimidoyl-α-D-glucopyranuronate, trimethylsilyl triflate, molecular sieves 4 Å, CH_2_Cl_2_, 0 °C, 3 h; (c) LiOH, MeOH/H_2_O, rt., 24 h

We commenced the synthesis of (−)-11-OH-Δ-9-THC-Glc (**1**), (2*S*,3*S*,4*S*,5*R*,6*S*)-3,4,5-trihydroxy-6-(((6a*R*,10a*R*)-1-hydroxy-6,6-dimethyl-3-pentyl-6a,7,8,10a-tetrahydro-6*H*-benzo[*c*]chromen-9-yl)methoxy)tetrahydro-2*H*-pyran-2-carboxylic acid, (Fig. 3) with the commercially available (−)-11-OH-Δ-9-THC (**2**), which was acetyl protected, by stoichiometric deprotonation of the phenolic proton with *n*-butyllithium and subsequent treatment with acetic anhydride. This led to (−)-11-OH-Δ-9-THC-OAc (**12**) in 90% yield which was further glucuronidated in a similar fashion to the Δ^8^ derivative **13**; however, TMSOTf was used as Lewis acid. The yield amounted to 37%. The final deprotection was carried out in water and methanol using lithium hydroxide as base to provide (−)-11-OH-Δ-9-THC-Glc (**1**) in 95% yield.

#### Synthesis of (−)-11-OH-Δ-9-THC-OAc (**12**)

1H NMR (500 MHz, CDCl3, 299 K) δ = 6.56 (d, J = 1.8 Hz, 1H), 6.42 (d, J = 1.8 Hz, 1H), 6.32–6.30 (m, 1H), 4.02 (brs, 2H), 3.13 (brd, J = 11.3 Hz, 1H), 2.52–2.47 (m, 2H), 2.31–2.21 (m, 5H), 1.97 (ddt, J = 12.7, 6.8, 2.1 Hz, 1H), 1.72–1.66 (m, 1H), 1.61–1.54 (m, 2H), 1.45–1.37 (m, 4H), 1.34–1.25 (m, 5H), 1.10 (s, 3H), 0.90–0.86 (m, 3H). 13C NMR (126 MHz, CDCl3, 299 K) δ = 169.0, 154.6, 149.4, 143.2, 138.5, 124.7, 115.5, 114.5, 114.2, 77.6, 67.3, 45.8, 35.54, 34.1, 31.6, 30.7, 27.6, 26.8, 24.6, 22.7, 21.4, 19.5, 14.1. HRMS (ESI) *m*/*z*: 395.2193 calcd. for C_23_H_32_O_4_Na+ [M+Na]^+^, found 395.2191.

#### Synthesis of (−)-11-OH-Δ-9-THC-Glc-OAc (**13**)

1H NMR (600 MHz, CDCl3, 299 K) δ = 6.54 (d, J = 1.7 Hz, 1H), 6.40 (d, J = 1.7 Hz, 1H), 6.30 (s, 1H), 5.31 (t, J = 9.5 Hz, 1H), 5.23 (t, J = 9.7 Hz, 1H), 5.10 (dd, J = 9.5, 7.8 Hz, 1H), 4.57 (d, J = 7.8 Hz, 1H), 4.21 (brd, J = 12.1 Hz, 1H) 4.00 (d, J = 9.8 Hz, 1H), 3.97 (brd, J = 12.0 Hz, 1H), 3.82–3.77 (m, 1H), 3.73 (s, 3H), 3.10 (brd, J = 11.3 Hz, 1H), 2.51–2.44 (m, 5H), 2.27 (s, 3H), 2.21–2.12 (m, 2H), 1.96–1.90 (m, 1H), 1.68–1.63 (m, 1H), 1.59–1.54 (m, 2H), 1.40 (s, 3H), 1.39–1.25 (m, 5H), 1.23–1.15 (m, 4H), 1.14–1.12 (m, 1H), 1.12–1.08 (m, 11H), 1.08–1.03 (m, 4H), 0.87 (t, J = 7.0 Hz, 3H). 13C NMR (150.73 MHz, CDCl3, 299 K) δ = 176.1, 175.4, 175.2, 169.0, 167.4, 154.6, 149.4, 143.2, 139.3, 134.4, 127.2, 115.5, 114.3, 114.1, 100.0, 77.5, 73.8, 73.0, 71.8, 70.9, 69.4, 52.9, 45.5, 35.5, 34.2, 34.0, 34.0, 31.6, 30.7, 27.5, 26.8, 24.5, 22.7, 21.3, 19.4, 19.0, 18.9, 18.9, 18.9, 18.8, 14.1. HRMS (ESI) *m*/*z*: 795.3926 calcd. for C_42_H_56_O_13_Na+ [M+Na]^+^, found 795.3928.

#### Synthesis of (−)-11-OH-Δ-9-THC-Glc (**1**)

1H NMR (600 MHz, CD3OD, 299 K) δ = 6.80 (d, J = 2.1 Hz, 1H), 6.18 (d, J = 1.7 Hz, 1H), 6.10 (d, J = 1.7 Hz, 1H), 4.35 (d, J = 7.9 Hz, 1H), 4.25 (d, J = 11.4 Hz, 1H), 4.11 (d, J = 11.4 Hz, 1H), 3.68 (d, J = 9.8 Hz, 1H), 3.52–3.48 (m, 1H), 3.41 (t, J = 9.1 Hz, 1H), 3.28–3.23 (m, 2H), 2.48–2.40 (m, 3H), 2.32–2.25 (m, 1H), 2.03–1.98 (m, 3H), 1.65 (td, J = 11.9, 11.1, 2.1 Hz, 1H), 1.60–1.54 (m, 2H), 1.08 (s, 3H), 0.96–0.90 (m, 4H). 1.47–1.28 (m, 10H). 13C NMR (150.73 MHz, CDCl3, 299 K) δ = 157.2, 155.8, 143.6, 134.5, 131.4, 109.7, 109.7, 108.4, 102.7, 78.0, 77.8, 76.3, 74.8, 74.8, 73.6, 47.2, 36.6, 35.2, 32.6, 32.0, 28.1, 28.0, 25.8, 23.6, 20.8, 19.4, 14.4. HRMS (ESI) *m*/*z*: 505.2443 calcd. for C_27_H_37_O_9_− [M−H]^−^, found 505.2438.

### In vitro analysis

The human liver S9 fraction assay generated phase I and phase II metabolites, and the positive paracetamol control was successfully glucuronidated. The negative control did not produce any metabolites as expected. The metabolites, detected with HPLC-HESI-HRMS in full scan MS-1 mode with a mass range from *m*/*z* 200 to 1000, are listed in Table 1. It shows HESI(+) data—sorted by calculated mass (*m*/*z*)—retention time (RT), chemical formula, adduct, calculated and found mass, and mass error in parts per million (ppm), and the three most abundant fragments of the detected metabolites. If available, the metabolites were verified with authentic standards. As identification criteria, we committed retention time errors below 0.1 min, mass errors less than 5 ppm, and consistent MS-2 spectra.

**Table 1:** Detected analytes (sorted by calculated mass) in the in vitro assay. The following parameters are given in this table: retention time (RT), chemical formula, adduct, calculated and found mass (*m*/*z*), mass error, and three most abundant fragment ions; *no MS-2 comparison, because no standard was available

Analyte	RT (min)	Chemical formula	Adduct	Calc. mass (*m*/*z*)	Found mass (*m*/*z*)	Mass deviation (Δppm)	3 most abundant fragments (*m*/*z*) (% rel. Int.)
(−)-Δ-9-THC	8.8	C_21_H_30_O_2_	[M+H]^+^	315.2319	315.2320	0.40	193.1227 (100) 135.1166 (37) 93.0703 (37)
8-OH-Δ-9-THC	5.0	C_21_H_30_O_3_	[M+H]^+^	331.2268	331.2266	− 0.60	201.0907 (100) 257.1529 (62) 81.0706 (46)
(±)-11-OH-Δ-9-THC	5.8	C_21_H_30_O_3_	[M+H]^+^	331.2268	331.2267	− 0.30	193.1225 (100) 201.0910 (87) 91.0548 (65)
(−)-Δ-9-THC-COOH	5.9	C_21_H_28_O_4_	[M−H]^−^	343.1903	343.1912	2.50	299.2007 (100) 245.1545 (37) 191.1066 (19)
(−)-Δ-9-THC-Glc	4.8	C_27_H_38_O_8_	[M+H]^+^	491.2639	491.2644	0.90	193.1228 (100) 315.2325 (75) 259.1693 (47)
G1*	2.5	C_27_H_38_O_9_	[M+H]^+^	507.2588	507.2592	0.66	209.1169 (100) 81.0706 (97) 93.0703 (81)
G2*	2.9	C_27_H_38_O_9_	[M+H]^+^	507.2588	507.2564	1.53	313.2168 (100) 193.1227 (46) 201.0906 (41)
*(−)-11-OH-Δ-9-THC-Glc (C11-O-Glc)*	3.4	C_27_H_38_O_9_	[M+H]^+^	507.2589	507.2604	3.00	313.2167 (100) 193.1227 (82) 217.1226 (76)
G3*	3.6	C_27_H_38_O_9_	[M+H]^+^	507.2588	507.2592	0.78	193.1228 (100) 313.2165 (76) 217.1226 (55)
G4*	5.0	C_27_H_38_O_9_	[M+H]^+^	507.2588	507.2589	0.19	135.1167 (100) 93.0703 (98) 209.1170 (86)
(−)-Δ-9-THC-COOH-Glc	2.8	C_27_H_36_O_10_	[M+H]^+^	521.2381	521.2389	1.70	299.2007 (100) 327.1955 (58) 193.1225 (48)

### Investigation of OH-THC metabolites

**Fig. 4:**
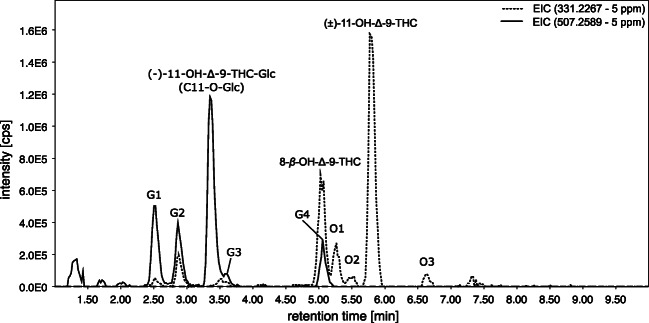
Extracted ion chromatogram (EIC) of *m*/*z* 331.2267 (5 ppm) for 11-OH-Δ-9-THC and *m*/*z* 507.2589 (5 ppm) for 11-OH-Δ-9-THC-Glc in heated electrospray ionization (HESI) positive mode of the sample obtained by the human liver S9 fraction assay

Focusing on OH-THC metabolites, in Fig. 4, the extracted ion chromatogram (EIC) in HESI positive mode of *m*/*z* 331.2276 (calculated exact mass for [M+H]^+^ of OH-THC) and *m*/*z* 507.2589 (calculated exact mass for [M+H]^+^ of OH-THC-Glc) is shown.

The EIC of *m*/*z* 331.2267 (dotted line) shows 11-OH-Δ-9-THC (RT 5.8 min), 8-OH-Δ-9-THC (RT 5.0 min), and the substances O1 (RT 5.3 min), O2 (RT 5.5 min), and O3 (RT 6.6 min). The metabolites O1-O3 are assumed to be OH-THC derivates, as the exact mass fits the OH-THC derivates and the HESI(+)-MS-2 spectra showed similar neutral losses as the known metabolites 11-OH-Δ-9-THC and 8-OH-Δ-9-THC. The biotransformation of different hydroxy-metabolites is in line with several studies of in vivo and/or in vitro hydroxylation of (−)-Δ-9-THC [4–6, 23–26]. In the range of 2 to 4 min, the signals can be assigned to in source fragments of OH-THC-glucuronides because they have the same retention time as the *m*/*z* 507.2589 of the glucuronides.

**Fig. 5:**
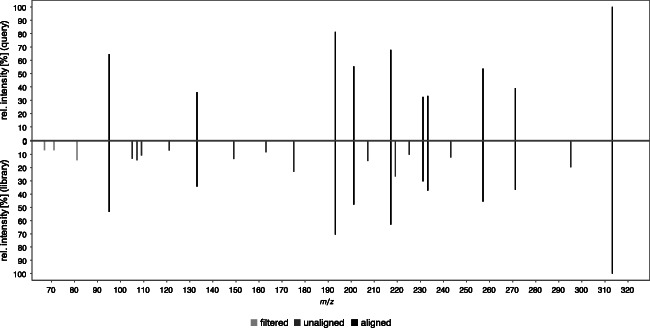
MS-2 spectrum pairing of precursor ion *m*/*z* 507 at retention time 3.4 min of the new synthesized reference standard ((−)-11-OH-Δ-9-THC-Glc; processed spectrum for database was used) and S9-assay with (−)-Δ-9-THC as substrate; weighted dot-product score: 0.918 (MassBank *m* = 2, *n* = 0.5 [27])

Figure 4 shows also glucuronides of OH-THC (calculated exact mass of [M+H]^+^: *m*/*z* 507.2589, black line). A total of 5 peaks at 2.5 min (G1), 2.9 min (G2), 3.4 min (11-OH-Δ-9-THC-Glc), 3.6 min (G3), and 5.0 min (G4) were detected. The comparison of the HPLC-HRMS data (retention time, exacted mass, and the fragment spectra) of the main peak at 3.4 min and the synthesized reference standard identified this as (−)-11-OH-THC, glucuronidated at the primary hydroxy group (hereafter referred to as alcoholic (−)-11-OH-Δ-9-THC-Glc). Figure 5 shows the MS-2 spectrum pairing, which yields a match of 91% [27]. Thus, the main peak at 3.4 min represents alcoholic (−)-11-OH-Δ-9-THC-Glc (1), synthesized in this study.

The other metabolites G1-G4, shown in Fig. 4, are assumed to also represent OH-THC-Glc metabolites, as the exact mass fits to OH-THC-Gluc, and their HESI(+)-MS-2 spectra all show the initial neutral loss of the glucuronic acid which leads to a fragment with *m*/*z* 331.2276. A comparison with the MS-2 spectrum of 11-OH-Δ-9-THC-Glc encourages this through the presence of most of the other fragments, but with different relative intensities.

### Investigation of inseparable (−)-11-OH-Δ-9-THC-Glc and G3

**Fig. 6:**
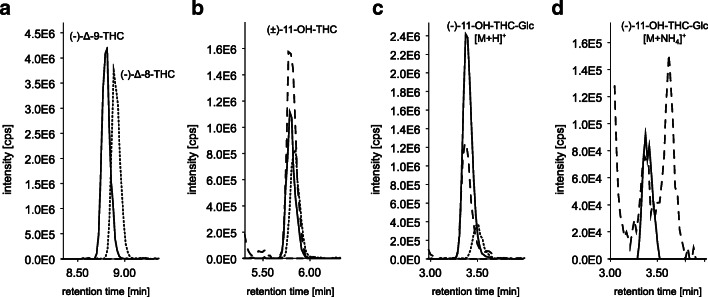
Extracted ion chromatograms (EIC) with a tolerance of 5 ppm of **a** THC, **b** 11-OH-THC, **c** (−)-11-OH-THC-Glc [M+H]^+^, **d** (−)-11-OH-THC-Glc [M+NH4^]+^; lines are the Δ-9 isomers, dotted lines are the corresponding Δ-8 isomers of the reference standards, and dashed line is the S9-assay with (−)-Δ-9-THC as precursor

The peaks of (−)-11-OH-Δ-9-THC-Glc and G3 (see Fig. 4) are only slightly separated chromatographically. Initial considerations suggested that G3 was the Δ-8 double bond isomer of (−)-11-OH-Δ-9-THC-Glc. A similar question was investigated for Δ-8 analogue of Δ-9-THC-COOH by Hanisch et al. [28]. In order to investigate the selectivity of our HPLC method with regard to Δ-8/ Δ-9 diastereomers, the previously synthetized (−)-11-OH-Δ-8-THC-Glc was used as reference substance. In Fig. 6, the extracted ion chromatograms in HESI positive of (A) THC *(m*/*z* 315.2318), (B) for OH-THC (*m*/*z* 331.2267), (C) for (−)-11-OH-THC-Glc (*m*/*z* 507.2588), and (D) (−)-11-OH-THC-Glc [M+NH_4_]^+^ (*m*/*z* 524.2854) are shown. For THC (graph A), (−)-Δ-8-THC (8.9 min) eluted slightly later than (−)-Δ-9-THC (8.8 min) in the chromatographic run. Thus, the peaks are not completely separated, similar to the separation of (−)-11-OH-Δ-9-THC-Glc and G3 in the in vitro assay. For 11-OH-THC (graph B), coelution of the isomers and the signal of the in vivo assay were observed. For (−)-11-OH-THC-Gluc (graph C), the Δ-8 isomer (RT 3.5 min) elutes as hypothesized slightly later than its Δ-9 isomer (RT 3.4 min), but the G3 signal of the in vitro assay elutes even later (RT 3.6 min). In addition to different retention times, we observed a different behavior in the adduct formation of the diastereomers. The Δ-9 isomer forms NH_4_^+^ adducts to a small extent, while the Δ-8 isomer does not form NH_4_^+^ adducts. Graph D shows no signal for the Δ-8 isomer (dotted line, 3.5 min), whereas the Δ-9 isomer (line, RT 3.4 min) and G3 signal (dashed line, RT 3.6 min) show NH_4_^+^ adduct formation. Altogether, due to retention time and adduct formation, the comparison of the G3 signal and the previously synthetized (−)-11-OH-Δ-8-THC-Glc and (−)-11-OH-Δ-9-THC-Glc reference standards leads to the conclusion that G3 is not (−)-11-OH-Δ-8-THC-Glc. Hence, there is no isomerization into Δ-8 isomer during in vitro metabolism. This interpretation is in line with Hanisch et al. [28] who concluded that this peaks does not represent Δ-8-THC-COOH but an artifact, which may arise from acyl migration of the corresponding phase II metabolite THC-COOH glucuronide [29].

### Investigation of glucuronide bounding

**Fig. 7:**
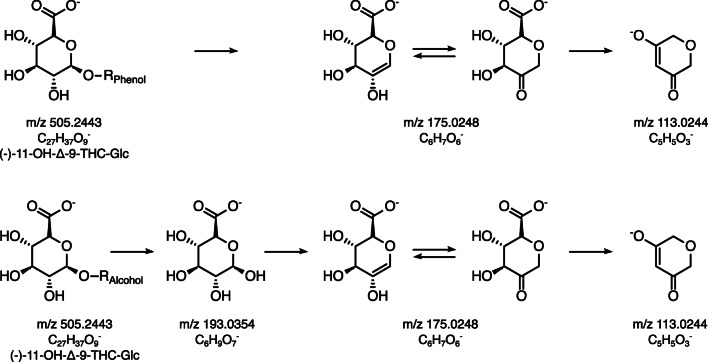
Proposed fragmentation pathway of phenolic and alcoholic glucuronides in negative ionization mode, modified from Wen et al. [30]

To determine whether the metabolites G1-G4 are alcoholic glucuronides (glucuronidation of the primary hydroxy group) or phenolic glucuronides (glucuronidation of the phenolic hydroxy group), we followed the approach described by Wen et al. [30]. They characterized phenolic and alcoholic structures in general and stated that in negative ionization mode, the presence of the fragments *m*/*z* 113, *m*/*z* 175, and *m*/*z* 193 is typical for alcoholic glucuronides, whereas the absence of fragment *m*/*z* 193 is typical for phenolic glucuronides (Fig. 7).

**Fig. 8:**
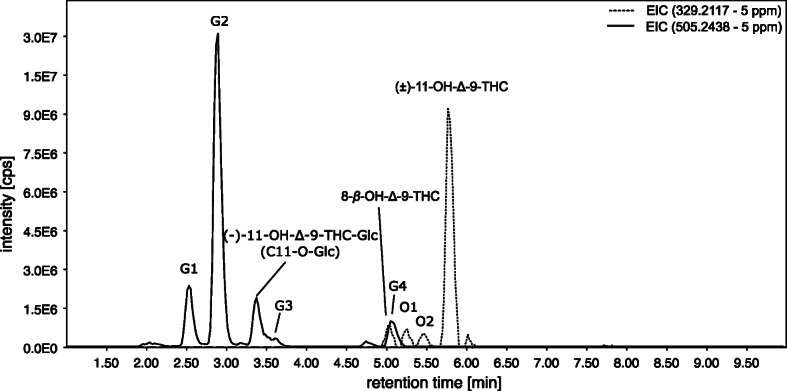
Extracted ion chromatogram of *m*/*z* 329.2117 (5 ppm) for OH-Δ-9-THC and *m*/*z* 505.2438 (5 ppm) for OH-Δ-9-THC-Glc in HESI negative mode of the S9-assay

In negative ionization mode (see Fig. 8), the EIC of OH-THC derivates (*m*/*z* 329.2117) and OH-THC-Glc (*m*/*z* 505.2438) shows peaks representing (±)-11-OH-Δ-9-THC (RT 5.8 min), 8-OH-Δ-9-THC (RT 5.0 min), as well as the substances O1 (RT 5.3 min) and O2 (RT 5.5 min). The small signal of O3 (RT 6.6 min) of HESI positive mode is missing in HESI negative mode, probably due to different ionization efficiency. For *m*/*z* 505.2438, the five glucuronides G1-G4 and (−)-11-OH-Δ-9-THC-Glc of the HESI positive analysis are also detected in HESI negative mode. The ionization efficiency of G2 and alcoholic (−)-11-OH-Δ-9-THC-Glc is different: in negative HESI mode G2 whereas in positive HESI mode alcoholic (−)-11-OH-Δ-9-THC-Glc presents the main signal. The reason for this effect is currently unclear to the authors.

**Fig. 9:**
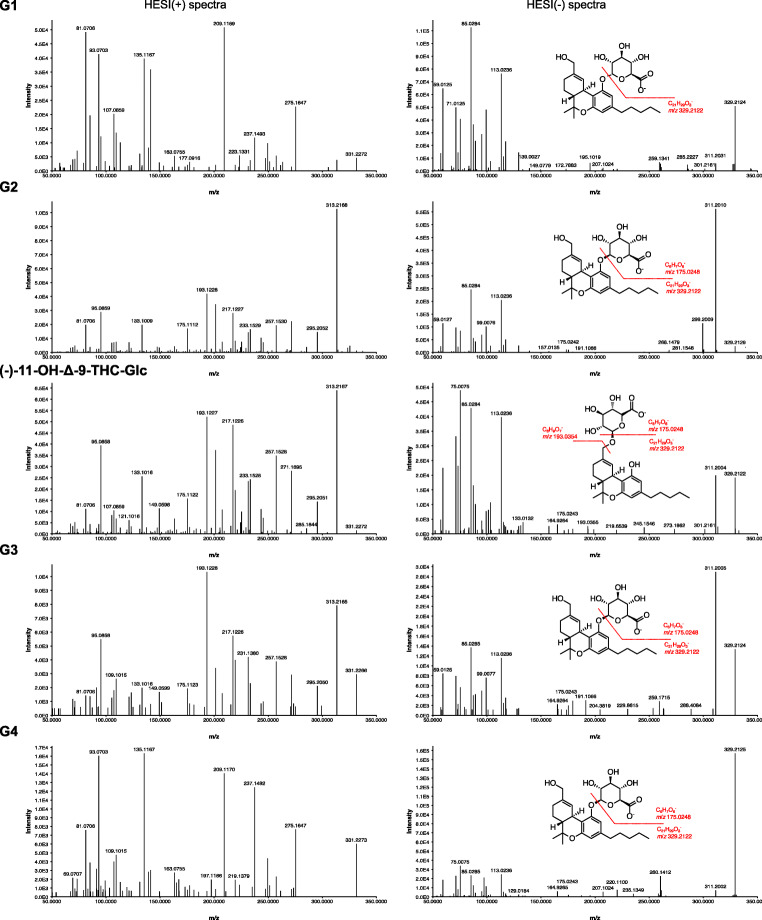
MS-2 spectra (*m*/*z* 50 to *m*/*z* 350) of the five OH-THC-Glc with *m*/*z* 507 in HESI positive mode and *m*/*z* 505 in HESI negative mode; proposed fragmentation pattern are indicated for differentiation of phenolic or alcoholic glucuronides

Following the abovementioned approach of Wen et al. [30], the MS-2 spectra (*m*/*z* 50 to *m*/*z* 350) of G1-G4 and (−)-11-OH-Δ-9-THC-Glc are analyzed in HESI positive (*m*/*z* 507) and negative (*m*/*z* 505) mode (see Fig. 9). For (−)-11-OH-Δ-9-THC-Glc, all three fragments (*m*/*z* 113, 175, 193) were present, which confirms its nature as an alcoholic glucuronide, which was already shown by the newly synthesized reference standard. However, it can be assumed that G1, G2, G3, and G4 are phenolic glucuronides, since the HESI (−)-MS-2 spectrum shows only the fragments *m*/*z* 113 and *m*/*z* 175 (also missing for G1 due to low concentration), whereas *m*/*z* 193 is missing. A verification of this hypothesis should be attempted once reference standards are available.

### Analysis of authentic specimen

**Fig. 10:**
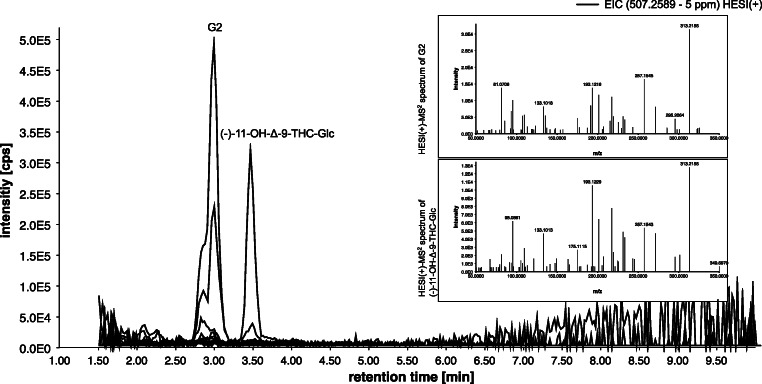
Extracted and overlaid ion chromatograms of *m*/*z* 507.2589 (5 ppm) in HESI(+) for OH-THC-Glc of ten authentic urine sample, analyzed by dilute and shoot approach; MS-2 spectra of the precursor 507 in HESI(+) for the corresponding peaks G2 (phenolic bound glucuronide) and (−)-11-OH-Δ-9-THC-Glc (alcoholic bound glucuronide)

To confirm the in vitro results, first ten urine samples of cannabis users were analyzed by dilute and shoot preparation and HPLC-HRMS in full scan mode. Figure 10 shows the overlaid EICs of OH-THC-Glc (HESI positive *m*/*z* 507.2588) of the ten urine samples. Only in two of the ten Δ-9-THC-COOH positive tested urines that OH-THC-glucuronides were detectable. Comparing in vitro data with in vivo urine data, it is noticeable that the urine samples contain only the two main metabolites of a total of five previously detected OH-THC-Glc of the in vitro assay. Diglucuronide of OH-THC was again not observed. In contrast to the in vitro assay, G2 is formed in urine in a greater extent than (−)-11-OH-Δ-9-THC-Glc. As described before, G2 metabolite seems to be a phenolic glucuronidated OH-THC, but a clear identification of this signal is still outstanding due to a lack of reference standards. The second main metabolite was successfully identified as the alcoholic (−)-11-OH-Δ-9-THC-Glc, confirmed by our novel reference standard.

The first positive urine sample belongs to a daily consuming person of medicinal cannabis. The second positive urine is from a person for checking fitness to drive with a concentration of 5.5 ng/mL (−)-Δ-9-THC and (−)-Δ-9-THC-COOH of 54 ng/mL in serum. The other eight negative samples for OH-THC-glucuronide have (−)-Δ-9-THC-COOH concentrations in serum between not detectable (only detectable in urine up to 20 ng/mL (−)-Δ-9-THC-COOH) and 26 ng/mL (−)-Δ-9-THC-COOH. These data indicate that OH-THC-glucuronides in urine only occur at higher concentrations of the phase I metabolites. This may be explained by the fact that 11-OH-THC is excreted mainly via the feces and less via the kidneys [23, 31].

**Fig. 11:**
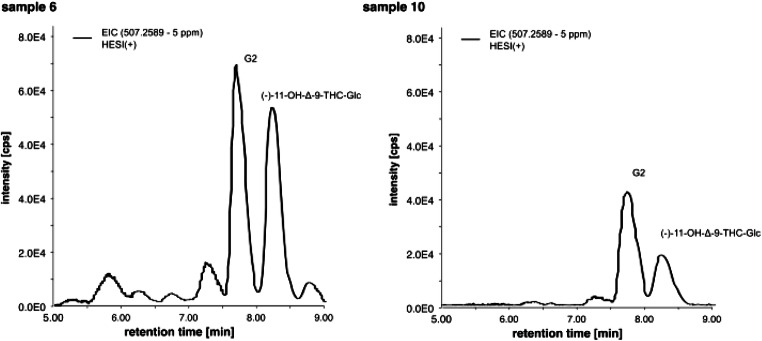
Extracted ion chromatograms of *m*/*z* 313.2162 (PRM mode, precursor ion *m*/*z* 507) in HESI(+) for OH-Δ-9-THC-Glc of two authentic serum samples extracted with a newly developed solid phase extraction method

Additionally, ten serum samples were analyzed after SPE for phase I/II metabolites by HPLC-HRMS in PRM mode and were identified and quantitated by reference standards. In contrast to urine samples, in eight of ten serum samples, OH-THC-glucuronides were detectable. In eight serum samples, the alcoholic (−)-11-OH-Δ-9-THC-Glc was successfully identified by reference standard. Quantification data for phase I/II-metabolites are listed in Table 2. The data suggest that the alcoholic (−)-11-OH-Δ-9-THC-Glc is not detectable at lower cannabinoid serum concentrations. Figure 11 shows to EICs of OH-THC-Glc of the samples with high (sample 6) and low (sample 10) concentrations of (−)-11-OH-Δ-9-THC-Gluc. In all positive (−)-11-OH-Δ-9-THC-Gluc samples, the chromatographic peak of the phenolic 11-OH-Δ-9-THC-Gluc (probably; G2) is higher than the chromatographic peak of the alcoholic (−)-11-OH-Δ-9-THC-Gluc, but the ratio seems to vary. In sample 6, the alcoholic (−)-11-OH-Δ-9-THC-Gluc peak is almost as high as the G2 peak, whereas in sample 10, the peak of alcoholic (−)-11-OH-Δ-9-THC-Gluc is only half the peak of G2.

**Table 2:** Concentrations (ng/mL) of main phase I/II THC-metabolites in serum after cannabis consumption. Lower limit of detection (LLOD) (−)-Δ-9-THC-Gluc = 0.10 ng/mL; (−)-11-OH-Δ-9-THC-Gluc = 0.05 ng/mL

Sample	1	2	3	4	5	6	7	8	9	10
(−)-Δ-9-THC	17	8.8	5.2	19	5.5	11	19	13	11	21
(−)-Δ-9-THC-Gluc	n.d.	n.d.	n.d.	n.d.	n.d.	n.d.	n.d.	n.d.	n.d.	n.d.
(−)-11-OH-Δ-9-THC	6.9	2.4	2.2	7.1	2.6	6.9	5.9	3.8	5.7	5.5
(−)-11-OH-Δ-9-THC-Gluc (alcoholic)	3.5	n.d.	n.d.	5.2	0.56	5.5	1.5	1.7	3.5	0.56
(−)-Δ-9-THC-COOH	64	54	83	50	44	218	266	39	29	248
(−)-Δ-9-THC-COOH-Gluc	ca. 285*	198	ca. 347*	ca. 281*	128	ca. 710*	ca. 1050*	297	151	ca. 680*

*Upper limit of quantification (ULOQ) (−)-Δ-9-THC-COOH-Gluc = 200 ng/mL)

## Conclusion

The investigation of phase II metabolism of 11-hydroxy-Δ-9-tetrahydrocannabinol reveals two main OH-Δ-9-THC-glucuronides in vitro and in vivo—an alcoholic and a presumably phenolic glucuronide. A double glucuronidation was not observed. The alcoholic (−)-11-OH-Δ-9-THC-Glc was successfully chemically synthesized and can now be used as reference standard. HPLC-HRMS data of this novel reference standard were successfully matched with the data of the in vitro and in vivo samples (urine/serum) and have thus confirmed the biotransformation of alcoholic (−)-11-OH-Δ-9-THC-Glc in vivo. The other main metabolite is assumed to be a phenolic glucuronide, due to detailed analysis of MS-2 spectra. Confirmation by synthesis of a reference standard is still pending.

The newly developed synthesis strategy of alcoholic (−)-11-OH-Δ-9-THC-Glc provides a simple and straightforward way for the synthesis as reference standard. Furthermore, the availability of a reference standard for alcoholic (−)-11-OH-Δ-9-THC-Glc offers the possibility for direct identification and quantification. After availability of the phenolic glucuronide besides the alcoholic glucuronide, it can be investigated, if there is a toxicogenetic influence, e.g., of polymorphic UGT 1A9 [32, 33], on the site and rate of glucuronidation (alcoholic/phenolic) of the 11-OH-Δ-9-THC.

## Electronic supplementary material

Supplementary 1 ^**1**^**H-NMR** (600 MHz, CD3OD, 299 K) of the synthesized (-)-Δ9-11-HO-THC-Glc (1) (DOCX 35 kb).

Supplementary 2 ^**13**^**C-NMR** (150.73 MHz, CDCl3, 299 K) of the synthesized (-)-Δ9-11-HO-THC-Glc (1) (DOCX 46 kb).
