# Supplementary material for: Investigation of phase II metabolism of 11-hydroxy-Δ-9-tetrahydrocannabinol and metabolite verification by chemical synthesis of 11-hydroxy-Δ-9-tetrahydrocannabinol-glucuronide
Source: Int J Legal Med. 2020 Aug 17;134(6):2105–19. doi: 10.1007/s00414-020-02387-w (PMC7578173; doi:10.1007/s00414-020-02387-w)
Supplement: Supplementary file 2 — 13C-NMR (150.73 MHz, CDCl3, 299 K) of the synthesized (-)-Δ9-11-HO-THC-Glc (1) (DOCX 46 kb). [file 414_2020_2387_MOESM2_ESM.docx]

Supplement 2:
